# Psychological interventions to improve pain, fatigue, anxiety, depression, and quality of life in children and adults with hypermobility spectrum disorders and Ehlers-Danlos syndrome: a systematic review

**DOI:** 10.1007/s00296-023-05503-2

**Published:** 2023-12-13

**Authors:** Natalie L. Clark, Gurvinder Singh Kainth, Melissa Johnson, Amar Rangan, Lucksy Kottam, Katherine Swainston

**Affiliations:** 1 South Tees Hospitals NHS Foundation Trust, Middlesbrough, UK; 2 The Mary Kinross Trust and RCS Chair, Department of Health Sciences and Hull York Medical School, University of York, Heslington, UK; 3 Faculty of Medical Sciences, Newcastle University, Newcastle Upon Tyne, UK

**Keywords:** Psychological intervention, Systematic review, Lived experiences, Hypermobility, Ehlers-Danlos syndrome

## Abstract

Hypermobility spectrum disorders (HSD) affect individuals across physical, psychological and social domains, making assessment and management difficult. Management for this condition primarily focuses on addressing the musculoskeletal complaints using physiotherapy rather than the additional manifestations such as fatigue, anxiety and depression. This systematic review aims to identify psychological interventions and assess whether they improve the lived experiences of individuals with HSD. It also aims to assess which psychological interventions were most effective, which symptoms were most effectively managed by a psychological intervention, and whether there were differences between children and adults. Studies were included if they were a randomised controlled trial or pre/post-test design, a sample of any age and clinical diagnosis of HSD (including Ehlers-Danlos syndrome), used a psychological intervention and assessed the effect of the intervention on lived experiences using appropriate outcome measures. Risk of bias was assessed using the Mixed Methods Appraisal Tool. The results were narratively synthesised. Six studies were included in the review, one isolated psychological intervention and five incorporated a psychological intervention within a multidisciplinary programme. The interventions predominantly aimed to reduce pain including intensity, interference, pain-related fear and catastrophising, with anxiety and depression, affect, daily living, fatigue also being evaluated. The most beneficial psychological interventions were those delivered alongside physiotherapy in an outpatient or community setting, improving both the physical and psychological aspects of pain, subsequently improving quality of life. However, there lacks randomised controlled trials with larger samples to definitively confirm the significant findings discussed in this review.

## Introduction

Joint hypermobility is defined as the ability to move a joint actively or passively beyond the normal physiological limits, seen as a symptom rather than an individual diagnosis [1]. It can be an indication of conditions such as Ehlers-Danlos Syndrome (EDS), specifically the hypermobile type, a heritable connective tissue disorder that can be diagnosed using the 2017 diagnostic criteria [1, 2]. Another associated condition is hypermobility spectrum disorders (HSD) [2]. This better classifies the disorders involving joint hypermobility across a spectrum, including asymptomatic joint hypermobility, which are diagnosed in the absence of meeting the hypermobile-EDS clinical criteria [2]. These two conditions (HSD and EDS) are often viewed as indistinguishable from one another.

Beyond the hypermobility symptom, additional physical manifestations of these conditions commonly include, musculoskeletal pain with intensity described ranging from tiring and exhausting to chronic and constant [3], fatigue with a prevalence of 77% [4], gastrointestinal symptoms such as abdominal pain (79%) and nausea (71%) [5], autonomic nervous system dysfunction [6]. Patients are more likely to experience anxiety and depression than the general population, with reported prevalence as high as 69% [3] and 75% [7] respectively. As a result of these physical and psychological manifestations, individuals exhibit social isolation behaviours [8], physical limitations in recreational activities [9], and employment difficulties [10]. The full biopsychosocial impact of these conditions has been comprehensively outlined within a recent scoping review [11].

The presenting complaints of those with HSD/EDS differ hugely from person to person, making an accurate diagnosis difficult. Assessment of patients requires a multifaceted approach and interdisciplinary collaboration, considering the physical and psychosocial (e.g., negative emotions, unhealthy patterns of activity) elements [12]. When this is neglected, patients can experience long, distressing diagnostic journeys with some patients reporting that it took 19 years to receive a diagnosis, despite their symptoms beginning in childhood [13]. Additional evidence found that healthcare professionals were more likely to dismiss patients if they stated their symptoms started from childhood [14]. Patients report consulting with healthcare professionals who lack knowledge and understanding of the condition, leading to many being misdiagnosed and mistreated, adding further unnecessary distress [14]. Once correctly diagnosed, however, it is important that the patient receives the most appropriate management for their condition.

Physiotherapy is frequently recommended for these patients, primarily to address their musculoskeletal complaints and joint pain. The success of physiotherapy however is variable, with one study reporting an improvement of 43% in patients and 38% reporting no improvement [15]. A qualitative study reported that attending physiotherapy can generate feelings of anxiety, or heighten a patient’s existing anxiety, as a result of a lack of awareness among their physiotherapists, previous negative interactions and exercises not being individualised [13]. A patient population such as this may benefit from management adopting a biopsychosocial approach, whereby psychological input could help address the psychopathological symptoms of pain, anxiety and depression, and benefit overall quality of life [16]. As an example, adaptations to existing physiotherapy programmes to ensure they are psychologically informed could offer a more holistic approach. Additional benefits of this individualised approach include helping to build a therapeutic alliance, setting goals and problem-solving, reconceptualising beliefs, fostering self-efficacy, and promoting self-management of their symptoms [17, 18]. Presently, there appears to be limited evidence on how to effectively manage patients beyond the obvious physical manifestations of HSD/EDS.

The primary aim is to systematically review whether psychological interventions improve the lived experiences of individuals with HSD/EDS. The secondary aims of the review include to determine: (1) which psychological interventions are most commonly used and most effective at improving the lived experiences of individuals with HSD/EDS; (2) which symptoms in individuals with HSD/EDS are most effectively managed by a psychological intervention; (3) whether there are differences between psychological interventions delivered for children/adolescents and adults with HSD/EDS.

## Methods

This review was pre-registered on prospero (CRD42022377904).

### Search strategy and selection criteria

The searches were conducted in December 2022 on seven databases, AMED, CINAHL, Cochrane Library, EMBASE, MEDLINE, PsycINFO, PubMed, supplemented by an additional grey literature source search on Google Scholar. The search terms for the strategy related to two keywords, hypermobility (including Ehlers-Danlos syndrome, hypermobility spectrum disorders, joint hypermobility, joint hypermobility syndrome, Ehlers-Danlos syndrome hypermobile type, hypermobile Ehlers-Danlos syndrome) and psychological interventions (including psychology, psychological, psychosocial, intervention, management, therapy). There was no restriction on the date of the publication though studies had to be available in full-text and in the English language.

Studies were included based on the following criteria: (1) study design: randomised controlled trials (RCTs) or pre-test/post-test; (2) sample with a clinical diagnosis of HSD or EDS of any age; (3) intervention: utilised and/or compared a psychological intervention; (4) psychological outcome measures (e.g., pain, quality of life).

### Screening

Retrieved articles were exported into the Rayyan referencing software to screen [19]. One author (NC) screened the titles and abstracts of the retrieved articles. Records were marked as “maybe” if eligible for a full-text review to be reviewed by a second author (GK), ineligible records at the title, abstract or full-text stage were marked as “excluded”. Any disagreements or uncertainties were resolved by a third author (KS), and eligible studies marked as “included”.

### Data extraction

Data were extracted by one author (NC). From the eligible studies, the following was recorded: author (year), country, participant characteristics (*N*, gender, age, diagnosis), intervention details (type, contents, length of intervention), outcome measures, results, conclusions, limitations and future directions. A meta-analysis was planned, however, due to the variation in outcome measures used, a narrative synthesis of the extracted data was undertaken instead.

## Results

**Fig. 1:**
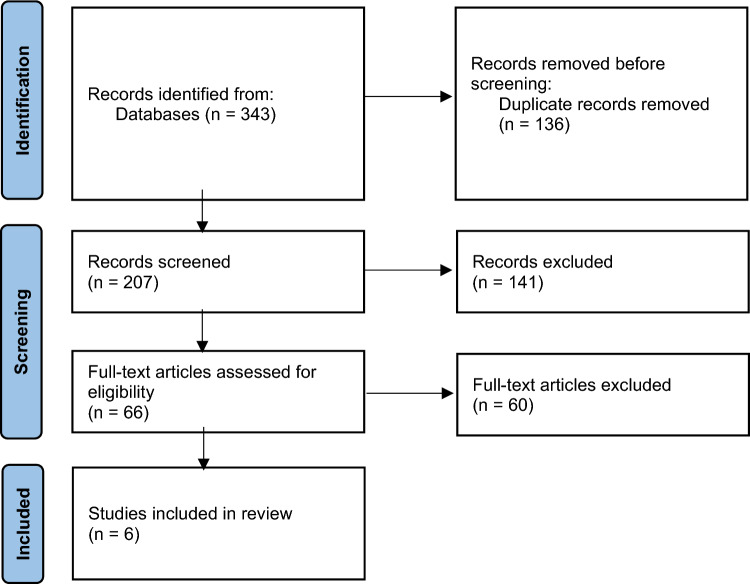
PRISMA flow diagram of the screening and selection process

The database search identified 343 records with no additional records identified through additional searches (e.g., hand search of reference lists). Following the removal of duplicates, 207 records remained for a title and abstract screening. The predominant reason for exclusion at this stage was the wrong study design (i.e., not RCT or pre-test/post-test). Following title and abstract screening, 66 records required a full-text review, with 6 meeting the inclusion criteria of this review. The flow diagram of the screening and selection process can be viewed in Fig. 1.

### Study characteristics

The total sample size of the six included studies was 628 however, due to attrition only 343 participants were analysed across the six studies. The sample was predominantly female (range 77.8–96.2%) with a mean age range of 14–39.2 years. The samples were mainly diagnosed with hypermobile-EDS [20–24]. Other diagnoses included generalised HSD [24], joint hypermobility syndrome [23, 25], classic EDS [21, 22], vascular EDS [22], other EDS [22]. Other subtypes of EDS were accepted for the review given the small proportion included and that HSD/hEDS still dominated the overall sample, this was therefore deemed permissible by the authors. The studies were conducted in the Netherlands [34], USA [23], UK [22, 25] France [21] and Italy [20] and were mostly pre-test/post-test [20, 21, 23–25] in design with one RCT [22]. Study characteristics of the included studies can be viewed in Table 1.

**Table 1:** Study characteristics

Author	Country	Aim	Study design	Sample characteristics				
				*N*	Female (%)	Mean age (SD)	Age range	Diagnosis (%)
Kalisch et al. [22]	UK	To examine the effects of the “Feeling Good Despite EDS” online intervention programme on positive and negative affect, pain interference, fatigue, life satisfaction, and satisfaction with the program in EDS patients	Randomised controlled trial	417 (132 analysed)	(96.2)	37.7	–	hEDS (84.8) cEDS (8.3) vEDS (2.3) other EDS (4.5)
van Meulenbroek et al. [24]	Netherlands	(1) To determine whether adolescents with G-HSD/hEDS showed changes in the level of disability after following MRT (2) To study whether improvements were found in physical functioning, perceived harmfulness and pain intensity in adolescents with G-HSD/hEDS after MRT	Pre-test post-test design	14	13 (92.9)	17.5	16.0 to 20.3	G-HSD/hEDS
Revivo et al. [23]	USA	To determine if paediatric patients with chronic pain related to JHS can be effectively treated with an interdisciplinary pain management program	Retrospective cohort study (pre-test post-test design)	30	27 (90)	14 (2.84)	9 to 18	hEDS (53.3) JHS (46.7)
Rahman et al. [25]	UK	To describe the intervention and the results of the programme	Pre-test post-test design	130	(96)	35	–	JHS
Chaleat-Valayer et al. [21]	France	(1) To describe this program (2) To evaluate the feasibility and efficiency of PrEduSED in terms of satisfaction and impact on disease management in daily life	Prospective observational study (Pre-test post-test design)	19	(89)	39.2 (15.2)	–	hEDS (89.5) cEDS (10.5)
Celletti et al. [20]	Italy	To evaluate a neurocognitive rehabilitation approach based on pain management and reduction as a primary outcome	Pre-test post-test design	18	14 (77.8)	21	13 to 55	hEDS

*cEDS* classical Ehlers-Danlos syndrome, *EDS* Ehlers-Danlos syndrome, *G-HSD* generalised hypermobility spectrum disorder, *hEDS* hypermobile Ehlers-Danlos syndrome, *JHS* joint hypermobility syndrome, *MRT *Multidisciplinary Rehabilitation Treatment, *PrEduSED *Programme d’Éducation thérapeutique des patients atteints du SED de type hypermobile, *SD *standard deviation, *UK *United Kingdom, *USA *United States of America, *vEDS *vascular Ehlers-Danlos syndrome

### Intervention characteristics

From the included studies, one study used a psychological intervention in isolation, with 5 studies incorporating a psychological intervention as part of a multidisciplinary programme. These are:

(1) A Positive Psychology Intervention programme for adults was delivered online over a 5-week period, evaluated by Kalisch et al. [22]. The intervention provided participants with five out of ten pre-determined positive psychology topics (spot the positives; mindful observation; savouring; socialising; a kindness day; self-compassion; using strengths in a new way; best possible self; gratitude visit; hope quest) to complete for 45 to 60 min per week. This was the only RCT of the included studies with three study groups: (1) assigned topics; (2) self-select topics; (3) waiting list (control). The intervention aimed to improve the wellbeing of patients with EDS, including emotions, fatigue, pain and life satisfaction.

(2) A Multidisciplinary Rehabilitation Treatment for adolescents delivered by a psychologist and physiotherapist over a 15-week period with the overall aim of the treatment to improve physical functioning and pain intensity, evaluated by van Meulenbroek et al. [24]. The first week was an introduction and education session with patients, followed by 8 weeks (2 h, twice a week) of physical therapy, aiming to improve physical parameters. The final 6 weeks (1 h a week) consisted of EXP therapy, aiming to restore a normal pattern of daily functioning.

(3) An Intensive Interdisciplinary Pain Management Programme for paediatric and adolescents delivered by a paediatric psychiatrist over a 4 to 8-week period (1 to 2 half-day sessions, per week), evaluated by Revivo et al. [23]. This programme delivered sessions on physical therapy (e.g., improving joint instability, strength and fitness), occupational therapy (e.g., pacing techniques), psychological interventions (e.g., self-management strategies) and medication management. Similarly, this aimed to improve physical functioning and pain.

(4) A Pain Management Programme for adults informed by a cognitive behavioural approach, delivered by a team of professionals including clinical psychologists, a nurse, physiotherapist and rheumatologists, evaluated by Rahman et al. [25]. This programme was delivered over 6 weeks (8 full days), covering pain beliefs, physical impact of pain, goal development and physiotherapy with the aim to improve pain.

(5) A Therapeutic Patient Education Programme consisted of ten workshops: me and my EDS; relaxation; the disease and my symptoms; pain medical treatment; how to move; administrative procedures and social rights; balneotherapy; activities of daily life; contention, orthosis and splits; psychological impact of hypermobile-EDS. The workshops were delivered during a 5-day hospital inpatient stay, evaluated by Chaleat-Valayer et al. [21]. The aim of this programme was to assess the impact of disease management in daily life.

(6) A Rehabilitation for Pain Management Programme, delivered by a therapist to patients of all ages used a neurocognitive behavioural approach over an 8-week period (60 min per week), evaluated by Celletti et al. [20]. The programme followed a rehabilitative plan, “felt sense” approach and language informed by narrative medicine. The aim of this programme was to assess its effectiveness in pain management and reduction.

### Risk of bias

The risk of bias was assessed using the Mixed Methods Assessment Tool [26] as it allows for the assessment of varied study designs, as included in this review. The tool uses two screening questions with follow-up questions depending on the study design. Two of the authors (NC, GK) completed the assessment. Three studies [21, 22, 24] were classified as moderate quality (40% to 60%) due to small sample sizes and incomplete datasets. Three studies [20, 23, 25] were of high quality (80%). See Table 2 for the summary.

**Table 2:** Risk of bias assessment

Study	Screening		Quantitative randomised controlled trials					Quantitative non-randomised					Total (%)
	Clear RQ	Data addresses RQ	Appropriate randomisation	Groups comparable at baseline	Complete outcome data	Outcome assessors blinded	Adhered to intervention	Representative of target population	Appropriate measurements	Complete outcome data	Confounders accounted for	Intervention administered as intended	
van Meulenbroek et al. [24]	Yes	Yes	–	–	–	–	–	No	Yes	No	Yes	Yes	60
Revivo et al. [23]	Yes	Yes	–	–	–	–	–	No	Yes	Yes	Yes	Yes	80
Kalisch et al. [22]	Yes	Yes	Unclear	Yes	No	Unclear	Yes	–	–	–	–	–	40
Rahman et al. [25]	Yes	Yes	–	–	–	–	–	Yes	Yes	No	Yes	Yes	80
Chaleat-Valayer et al. [21]	Yes	Yes	–	–	–	–	–	No	Yes	No	Yes	Yes	60
Celletti et al. [20]	Yes	Yes	–	–	–	–	–	Yes	Yes	No	Yes	Yes	80

*RQ* research question

### Main results

In line with the primary research questions, the main results discuss how psychological interventions improved the lived experiences of individuals with HSD/EDS. This included their psychological health, daily living, and symptoms such as pain and fatigue. Additional considerations from a relative’s perspectives and satisfaction with the intervention were also acknowledged within the results. The outcome measures used to assess these results were recorded, see Table 3.

**Table 3:** Outcome measures used by the study

Outcome measure	Assessing	Subscales/domains	Scoring
**Kalisch et al. **[22]**—positive psychology intervention (baseline, post-intervention and 1-month follow-up)**			
Client satisfaction questionnaire (CSQ-I)	Rating the intervention	8 items	• 4-point Likert scale: “does not apply to me” to “does totally apply to me” • Higher scores = higher satisfaction
Pain disability index (PDI)	Pain interference	7 items	• 11-point Likert scale: 0 (no disability) to 10 (worst disability) • Scores are summed, total score range 0 to 70 • Higher scores = worst disability
Positive and negative affect (PANAS)	Positive and negative affect in the past week	• 10 items for positive • 10 items for negative	• 5-point Likert scale: 1 (very slightly or not at all) to 5 (extremely) • Higher scores = greater agreement
Satisfaction with life scale (SWLS)	Life satisfaction	5 items	• 7-point Likert scale: 1 (strongly disagree) to 7 (totally agree) • Scores: o 5–9 = Extremely dissatisfied o 10–14 = Dissatisfied o 15–19 = Slightly dissatisfied o 20 = Neutral o 21–25 = Slightly satisfied o 26–30 = Satisfied o 31–35 = Extremely satisfied
Satisfaction with week’s exercises	Rating the intervention	N/A	• 5-point Likert scale: 1 (I didn’t enjoy them at all) to 5 (I enjoyed them very much) • Higher scores = greater agreement
Visual rating scale	Fatigue	N/A	• 11-point Likert scale: 0 (no disability) to 10 (worst imaginable fatigue) • Higher scores = worst disability
**van Meulenbroek et al. **[24]**—multidisciplinary rehabilitation treatment (baseline and post-intervention)**			
Functional disability inventory (FDI)	Perceived difficulty in performing activities at school, at home, in recreational or social interactions in adolescents	15 items	• 5-point Likert scale: 0 (no trouble) to 4 (impossible) • Scores: o 0–12 = no/minimal disability o 13–29 = moderate disability o 30 + = severe disability
Photograph series of daily activities for youth (PHODA-youth)	Perceived harmfulness	51 age-specific photographs: • Activities of daily living and household (13 items) • Intensive physical activities (27 items) • Social activities (11 items)	• 11-point Likert scale: 0 (not harmful at all) to 10 (extremely harmful) • Higher scores = higher perceived harmfulness
Visual Analog Scale (VAS)	Pain intensity	3 items: • Current pain • Worst/most severe pain experienced in the last week • Least pain experienced in the last week	• 100 mm line: no pain to worst pain imaginable
Revivo et al. [23]—Intensive Interdisciplinary Pain Management Programme (baseline and post-intervention)			
Bath adolescent pain questionnaire (BAPQ)	Chronic pain in patients aged 8 to 18	7 subscales: 1. Daily functioning (social and physical) 2. Emotional functioning (depression, general anxiety, pain specific anxiety) 3. Family functioning 4. Developmental functioning	• 5-point Likert scale: 0 (never) to 4 (always) • Higher scores = more impaired functioning
Bath adolescent pain-parent impact questionnaire (BAP-PIQ)	Chronic pain impact on parents	8 subscales: 1. Parental emotional functioning (depression and anxiety) 2. Catastrophic thinking about one’s child 3. Self-blame 4. Hopelessness 5. Relationship with partner 6. Leisure functioning 7. Parental behaviour 8. Parental strain	• 5-point Likert scale: 0 (never) to 4 (always) • Higher scores = more impaired function
Numeric Rating Scale (NRS)	Pain intensity	N/A	• 11-point Likert scale: 0 (no pain) to 10 (worst pain imaginable)
**Rahman et al. (2014) **[25]**—pain management programme (baseline, 1-month follow-up and 5-month follow-up)**			
Brief pain inventory (BPI)	1. Pain intensity 2. Impact of pain on life	9 items	• 11-point Likert scale: 0 to 10 • Average score = impact score • Higher scores = excruciating pain
Depression, anxiety and positive outlook scale (DAPOS)	Mood in pain patients	11 item subscales: 1. Depression (five items) 2. Anxiety (three items) 3. Positive outlook (three items)	N/S
Pain catastrophising scale (PCS)	A patient’s tendency to catastrophise	13 items	• Higher scores = higher thoughts and feelings when experiencing pain
Pain self-efficacy score (PSEQ)	Confidence in doing activities despite pain	10 questions	• Higher scores = greater levels of confidence in dealing with pain
**Chaleat-Valayer et al. **[21]**—therapeutic patient education programme (baseline and 6-month follow-up)**			
Coping strategies questionnaire—French version (CSQ-F)	Pain coping strategies	21 items, 5 factors: 1. Distraction 2. Catastrophising 3. Ignoring pain sensations 4. Reinterpreting pain sensations 5. Praying	• Higher scores = higher frequency of the strategy being used
Fatigue impact scale (FIS)	Functional limitations of fatigue over the past month	40 items: 1. Cognitive functioning 2. Physical functioning 3. Psychosocial functioning	• Higher score = the better the patient feels
Hardness scale (Zarit)	Carers burden	22 items: 1. Impact of the disease on the quality of life of the relative 2. Psychological and moral suffering 3. Financial difficulties 4. Shame 5. Difficulties in social and family relations 6. Guilt	N/S
Hospital anxiety and depression scale (HADS)	1. Body perceptions 2. Psychic feelings 3. Depression and anxiety	14 items: 1. Anxiety subscale 2. Depressive subscale	• 4-point Likert scale: 0 to 3
Questionnaire d’image du Corps (QIC)	Body perceptions	19 expressions	• 5-point Likert scale: 1 to 5 • Total score = 19 to 95 • Higher scores = higher body satisfaction
Social functions questionnaire (SF-12)	1. Quality of life 2. Impact of disease on daily activities	12 questions: 1. Physical functioning 2. Overall health-related quality of life	• Higher scores = better physical and mental health functioning
**Celletti et al. **[20]**—rehabilitation for pain management programme (baseline and post-intervention)**			
Fatigue severity scale (FSS)	Fatigue intensity	9 items	• 7-point Likert scale • Higher scores = higher severity of fatigue and effect on a person’s activities and lifestyle
McGill pain questionnaire	Pain intensity	3 subscales: 1. Sensory qualities 2. Affective qualities 3. Intensity of pain	N/S
Numeric rating scale (NRS)	Pain intensity	N/A	• 11-point Likert scale: 0 (no pain) to 10 (acute pain)
Oswestry disability index (ODI)	Pain and daily activity	10 questions: 1. Pain intensity 2. Personal care 3. Lifting 4. Walking 5. Sitting 6. Standing 7. Sleeping 8. Sex 9. Social 10. Travel	• 6-point Likert scale • Higher scores = higher pain-associated disability
Tampa scale of Kinesiophobia (TSK)	Pain and pain-related fear	2 subscales, 17 items: 1. Activity avoidance 2. Harm	• Higher scores = increasing degree of kinesiophobia

#### Pain

All six studies aimed to determine the effectiveness of their interventions on various characteristics of pain, predominantly in terms of pain intensity and interference. Revivo et al. [23] assessed the impact of chronic pain generally in adolescents using a sample-specific questionnaire, the Bath Adolescent Pain Questionnaire [27]. The questionnaire has a number of subscales including emotional (depression, general anxiety and pain-specific anxiety, daily (social and physical), family, and developmental functioning. Significant improvements in functioning were found post-intervention compared to baseline across all subscales (*p* < 0.05) with the exception of the latter two key adolescent features (*p* = 0.236 and *p* = 0.101 respectively). Family functioning, however, had unexpectedly improved for adolescents with EDS but not for those with JHS. It was unclear why this was found and warrants further exploration.

Chaleat-Valayer et al. [21] was the only study to measure adult’s pain coping strategies across five subscales [28]. However, no significant improvements were found in distraction (*M* = 13.0 vs 12.6, *p* = 0.581), reinterpretation (*M* = 3.6 vs 8.6, *p* = 0.502), ignorance (*M* = 11.5 vs 11.7, *p* = 0.878), dramatisation (*M* = 8.7 vs 7.9, *p* = 0.369), or prayer (*M* = 4.8 vs 4.6, *p* = 0.843) from baseline to 6-months follow-up. This suggests that a short intervention cannot properly address and deliver the coping strategies needed for this complex condition. However, given that the intervention was defined as one that should enable a patient to develop coping skills, a significant improvement in scores would have been expected.

##### Pain intensity

Four studies with both adolescent and adult patient samples assessed pain intensity using a variety of outcome measures, McGill Pain Questionnaire [29] in Celletti et al. [20]; Numeric Rating Scale (NRS) in Revivo et al. [23]; Visual Analog Scale in van Meulenbroek et al. [24]; Brief Pain Inventory (BPI) [30] in Rahman et al. [25]. All outcome measures have been widely used in the literature with good reliability and validity, with the NRS and BPI being validated for adolescent use. Pain intensity significantly improved from baseline scores compared with the post-intervention score (*p* ≤ 0.05) across all four studies, with one intervention reducing pain intensity in adolescent patients by 63% (*Mdn* = − 26.0, *p* = 0.005) [24]. Similarly, Revivo et al. [23] demonstrated clinically significant reductions in 36.7% of their adolescent sample, though notably 30% were reliably worse and 33.3% had no change. Nevertheless, this significant finding is noteworthy given the length of time some of adolescents had experienced pain symptoms for. Rahman et al. [25] were the only one of the four to measure the long-term effect of the intervention on pain intensity specifically in adult patients, with a follow-up assessment at 5 months. However, no significant difference was found when compared to baseline (*M* = 6.5 vs 6.4, *p* = 0.138). The four interventions were effective at reducing pain intensity and significantly more effective for adolescent patients than adults. In the absence of long-term follow-up in an adolescent sample though, it is difficult to definitively conclude this.

##### Pain interference

Pain interference refers to how pain can impact the ability of adolescents and adults to function daily. This was assessed by four studies [20, 22, 24, 25], using a variety of outcome measures, all with good reliability and validity. Kalisch et al. [22] used the Pain Disability Index [31] with an adult sample at baseline, post-intervention and 1-month follow-up, finding only a small improvement in post-intervention scores, with follow-up scores worse than baseline across all groups (*F*(2, 101) = 3.631, *p* < 0.05; partial η^2^ = 0.067). Pain disability was also measured by Celletti et al. [20] using the Oswestry Disability Index [32] and van Meulenbroek et al. [24] using the Functional Disability Inventory (FDI) [33]. Both studies found significant differences (*M* = 16 vs 10, *p* ≤ 0.001; *Mdn* = − 16.0, *p* = 0.001 respectively) between baseline and post-intervention. The FDI has been specifically noted as a valid and reliable measure for adolescent use. Finally, Rahman et al. [25] measured whether the intervention improved the patient’s confidence in participating in daily activities despite pain via the Pain Self-Efficacy Questionnaire [34]. A significant improvement of 27% was reported post-intervention (*M* = 25.3 vs 32.2, *p* < 0.001), which was also significantly sustained at 5-month follow-up (*M* = 28.2, *p* ≤ 0.002). These findings suggest a positive psychological intervention was the least effective at reducing pain interference in adult patients when compared to the three multidisciplinary interventions delivered to both adolescents and adults, with one demonstrating significant long-term improvements [25].

#### Psychological health

Four studies measured the impact the interventions had on psychological health, including pain-related fear and pain catastrophising, depression, anxiety, and positive and negative affect.

##### Fear and catastrophising

This population reports emotional responses to pain, including pain-related fear of movement and pain catastrophising. Celletti et al.’s [20] intervention helped to raise awareness amongst adolescents and adults of their movements, which in turn significantly reduced their fear linked to the movement from baseline to post-intervention (*M* = 34 vs 30, *p* ≤ 0.001). This was measured by the Tampa Kinesiophobia Scale [35], the most widely used scale for assessing pain-related fear. However, this intervention was specific to patients with chronic lower back pain and is therefore not generalisable to the wider HSD/EDS patient population. Catastrophising is a complex, cognitive distortion that can be influenced by psychological and physical factors and can be measured using the Pain Catastrophising Scale [36]. Rahman et al.’s [25] intervention successfully and significantly improved catastrophising in adult patients by 31.8% (*M* = 27.9 vs 19.0, *p* < 0.001), which was also significantly sustained at 5-month follow-up (*M* = 21.5, *p* < 0.001). This factor was the most improved in this study, suggesting this intervention was better at addressing pain-catastrophising though this was the only study that measured this factor.

##### Mood, anxiety and affect

Anxiety and depression are frequently reported in patients with HSD/EDS, though only two studies assessed whether the interventions were able to improve these factors. Rahman et al. [25] used the Depression, Anxiety and Positive Outlook Scale [37], comprising of these three subscales. Both depression and anxiety were significantly improved from baseline to post-intervention scores by 15.2% (*M* = 12.7 vs 10.7, *p* < 0.001) and 15.1% (*M* = 7.76 vs 6.6, *p* < 0.001) respectively, and significantly sustained at 5-months follow-up (*M* = 11.9, *p* = 0.015; *M* = 7.1, *p* = 0.013). Positive outlook was seemingly not measured. Alternatively, Chaleat-Valayer et al. [21] used the Hospital Anxiety and Depression Scale (HADS) [38] to assess these two factors, a frequently used and reliable scale. Pre-intervention, patients reported high anxiety and depression scores that the intervention was unsuccessful at significantly improving by 6-month follow-up (*M* = 10.8 vs 10.2, *p* = 0.655; *M* = 6.7 vs 7.2, *p* = 0.739 respectively). Notably, across the two studies, both depression and anxiety had only small changes, suggesting the multidisciplinary interventions were not successful in making improvements to these factors in adult patients.

In addition to anxiety and depression, affect was considered by Kalisch et al. [22] using the Positive and Negative Affect Schedule [39] to assess affective feelings such as interest and guilt. Only adult patients in the group that had self-selected their positive psychology topics had significantly higher levels of positive affect post-intervention which were also maintained at the 1-month follow-up (*F*(2, 101) = 5.839, *p* < 0.01; partial η^2^ = 0.104), with no significant improvements to negative affect across the 3 study groups at any timepoint (*F*(2, 101) = 1.007, *p* > 0.05; partial η^2^ = 0.020).

#### Daily living

Daily living was assessed in adult patients across two studies, Kalisch et al. [22] using the Satisfactions with Life Scale [40] and Chaleat-Valayer et al. [21] using the Social Functions Questionnaire (SF-12) [41], measuring quality of life by physical and mental functioning subscales. Similar to positive affect, patients within the self-selected group had a significantly higher satisfaction with life post-intervention and also at 1-month follow-up (*F*(2, 101) = 4.916, *p* < 0.01; partial η^2^ = 0.089) [22]. However, the intervention within Chaleat-Valayer et al.’s [21] study, like anxiety and depression, did not significantly improve the physical and mental functioning of adult patients from baseline to 6-month follow-up (*M* = 30.1 vs 31.0, *p* = 0.925; *M* = 42.9 vs 40.7, *p* = 0.661, respectively). Similar to the lack of significance in pain coping strategies, the short timeframe of this inpatient intervention is likely to be responsible for this finding.

#### Fatigue

Another common symptom of these conditions is fatigue. Despite this, only three studies assessed the impact of the intervention on fatigue. Chaleat-Valayer et al. [21] used the Fatigue Impact Scale [42] to measure functional limitations of fatigue across four subscales with a significant difference only found in one at the 6-month follow-up, cognitive (*M* = 16.4 vs 21.8, *p* = 0.127), physical (*M* = 11.9 vs 18.2, *p* = 0.08), social (*M* = 22.8 vs 26.3, *p* = 0.374), and relationship (*M* = 6.1 vs 8.1, *p* = 0.05). Celletti et al. [20] used the Fatigue Severity Scale [43] to quantify the intensity of fatigue, demonstrating a significant reduction pre- and post-intervention (*M* = 46 vs 40, *p* ≤ 0.05). The third study [22] simply asked patients to rate their fatigue on a scale from “no disability” to “worst imaginable”, with a small difference observed at 1-month follow-up that did not reach statistical significance (*F*(2, 101) = 2.141, *p* > 0.05; partial η^2^ = 0.041). These findings demonstrate that Celletti et al.’s [20] multidisciplinary intervention was more effective at improving fatigue, specifically the intensity, in both adolescents and adults than an inpatient intervention and an isolated psychological intervention.

#### Intervention satisfaction

Only one study [22] explored how satisfied patients were with the intervention. Overall, the majority of patients (76.6%) were either satisfied or very satisfied with the positive psychology intervention, with less than 5% not satisfied at all, and no significant differences in satisfaction levels between the two intervention groups. Patients from both intervention groups were asked to rate the individual 10 positive psychology topics using a 5-point Likert scale from 1 (I didn’t enjoy them) to 5 (I enjoy them very much). “Spot the positives” received the highest rating (*M* = 4.24) and “hope quest” received the lowest (*M* = 3.12). Patients in the self-selected group would choose “self-compassion” most often (77.8%), with “gratitude visit” less frequently chosen (16.7%).

#### Relative perspectives

Of the three studies including adolescents within their samples, two also assessed the parent’s perspective on how successful the intervention had been on the patient. Revivo et al. [23] used the Bath Adolescent Pain-Parent Impact Questionnaire [44] a reliable measure to assess the parental functioning of those with adolescents with chronic pain. It measures functioning across eight subscales with reductions in all pre- and post-intervention, though not significantly reduced in the latter two subscales: depression (*M* = 12.31 vs 7.73, *p* < 0.001), anxiety (*M* = 8.96 vs 5.73, *p* = 0.001), child-related catastrophising (*M* = 9.23 vs 5.65, *p* < 0.001), self-blame and helplessness (*M* = 11.81 vs 6.77, *p* < 0.001), partner relationship (*M* = 9.63 vs 8.37, *p* = 0.09), leisure functioning (*M* = 14.96 vs 11.92, *p* = 0.005), parental behaviour (*M* = 27.0 vs 19.27, *p* < 0.001), and parental strain (*M* = 8.12 vs 7.23, *p* = 0.252). This finding is particularly important as it demonstrates the role of parents in the management of HSD/EDS in adolescent patients.

Chaleat-Valayer et al.’s [21] study asked relatives to complete the SF-12 and HADS alongside the patients. These relatives were predominantly male with a mean age of 44.1 years. The validated Zarit Scale was also used to measure the quality of life of the relatives, specifically the burden felt across, psychological and moral suffering, financial, social and family difficulties, shame, and guilt domains. The overall Zarit score did not significantly change (*M* = 19.0 vs 21.4, *p* = 0.949). However, this lack of significant finding can be attributed to the majority of relatives (60%) considering there to be a light burden or no burden at all.

## Discussion

This systematic review has narratively synthesised the evidence on the use of psychological interventions to improve the lived experiences and symptoms of individuals with HSD/EDS. We identified six studies that used either an isolated psychological intervention or incorporated one within a multidisciplinary programme to address outcomes such as pain (intensity, interference, fear and catastrophising), fatigue, anxiety, depression, positive and negative affect, and quality of life.

The results confirmed the primary aim, in that psychological interventions are successful at making significant and sometimes even long-term improvements in the lived experiences of individuals with HSD/EDS, particularly within the pain domain. Pain in this patient population has been reported to affect the entire body, with an increased likelihood of also being diagnosed with a comorbid psychiatric disorder [11, 45]. In addressing the secondary aims of the review, the most beneficial interventions were found to be those that incorporated a psychological intervention alongside physical therapy [23–25]. These interventions significantly reduced the psychological (pain-related fear and catastrophising) and physical (functioning and disability) impact of pain in patients with HSD/EDS. Previous evidence supported that interventions targeting pain catastrophising and aiming to increase physical activity improves the outcomes in adult patients with chronic musculoskeletal pain [46], as found within an included study measuring catastrophising [25]. By comparison, an isolated, positive psychological intervention [22] could only improve the physical disability as a result of pain in the short term and was seemingly better at improving the affective factors in the long term. Furthermore, this combined psychological intervention and physical therapy design was effective for reducing pain interference and pain-related fear in both adolescents and adults [24, 25].

Despite small improvements across a number of factors (anxiety, quality of life, pain coping strategies and fatigue), the 5-day inpatient intervention [21] was unable to demonstrate sustained, significant improvements. In addition, it did not assess patients at discharge making it difficult to conclude if there were any significant improvements at least in the short term. The other included interventions were conducted in outpatient/community settings, whilst demonstrating significant findings. It would therefore seem to suggest that an inpatient intervention is not necessary or cost-effective and would indeed be costly. Notably within Kalisch et al. [22], the patients that were actively involved in the design of their intervention exhibited better outcomes. Patients who actively participate in the management of their conditions have been evidenced to become empowered, and when combined with multidisciplinary management can prevent absence from employment, reduce associated healthcare costs, and increase health-related quality of life [47].

Our findings confirm the previous suggestions to develop and use psychologically informed physiotherapy approaches, especially for pain [17], would be welcomed by both patients and healthcare professionals [48]. However, for this to be effective, there needs to be an increase in psychological intervention training and knowledge for physiotherapists, including cognitive behavioural therapy, effective communication, and behaviour change techniques [48]. Recommended and successful behaviour change techniques for HSD/EDS, have been those that aim to reduce pain-related fear and catastrophising [49] and have been evidenced as successful in the interventions in the present review. It was not evident whether the psychological interventions identified within the review were informed by theoretical approaches such as the Theoretical Domains Framework or the Capability, Opportunity, Motivation-Behaviour model [49].

### Limitations

There are a few limitations of this review. Firstly, half of the included studies had a moderate risk of bias, predominantly due to small sample sizes and incomplete outcome data as a result of attrition at follow-up. Secondly, the review was not able to complete a meta-analysis as per protocol due to heterogeneous outcomes used and measured, therefore would not have accurately quantified the impact of the interventions on the lived experiences of this patient population. Lastly, there were a small number of included studies, with small sample sizes, and only one being an RCT. It is, therefore, difficult to definitively state the effectiveness of the interventions and whether these can be implemented in this patient population in the absence of adequately powered randomised trials.

## Conclusions

Irrespective of the age of the patient, the most effective interventions for HSD/EDS were those that were multidisciplinary and targeted the physical and psychological impact of pain and physical disability. Addressing these factors will in turn improve additional symptoms of HSD/EDS, such as fatigue, depression and anxiety, and quality of life. It is important for healthcare professionals and patients to work in collaboration to ensure the intervention is designed and tailored appropriately for the patient and their presenting complaints, as informed by suitable theoretical approaches. Future research should attempt to replicate the findings of the pre-test/post-test interventions using adequately powered RCTs and a longer-term follow-up period to confirm the effectiveness of the interventions to establish whether lasting improvements are possible.

## Data Availability

The data that supports the findings of this review are available from the corresponding author upon reasonable request.

## Abbreviations

BPI: Brief Pain Inventory
EDS: Ehlers-Danlos syndrome
FDI: Functional Disability Index
HSD: Hypermobility spectrum disorders
M: Mean
MDN: Median
NRS: Numeric rating scale
RCT: Randomised controlled trial
UK: United Kingdom
USA: United States of America
